# Supplementary material for: Post lockdown COVID-19 seroprevalence and circulation at the time of delivery, France
Source: PLoS One. 2020 Oct 15;15(10):e0240782. doi: 10.1371/journal.pone.0240782 (PMC7561105; doi:10.1371/journal.pone.0240782)
Supplement: Attachment — Submitted filename: Review of Post lockdown COVID.docx [file pone.0240782.s002.docx]

“Post lockdown COVID-19 seroprevalence and circulation at the time of 1

delivery, France” reported that the positive rates of SARS-COV2 IgG and virus RNA of throat samples in group of patients in maternity ward around the end of LOCKdown timepoints. Although the data is convincing, it does not look conclusive since no data was shown the positive rates before the end of lockdown(ie. 14 days before lockdown; lockdown periods, postlockdown). Secondly, except that the only diagnosed patients were both RNAand IgG positive and had symptoms and epidemiological evidence, no current infection of SARS-COV-2 were tested for all other patients since no IgM test were performed(sensitivity of RNA detection is usually low). So , the conclusion that the virus was no longer circulating up to three weeks after the end of the lockdown may not be acurate. Thirdly. “ half of the SARS-CoV-2-IgG-positive pregnant women were completely asymptomatic “may not be acurate since a longer time (more than 5 months )of history of symptoms may be required. Therefore I do not think the manuscript merit to publish in Plos one Journal unless these issues are resolved. However, the conclusion that “a high proportion of SARS-CoV-2-IgG-negative pregnant women must be taken into into consideration in the event of a resurgence of the pandemic...” is helpful.
