# Supplementary material for: Post lockdown COVID-19 seroprevalence and circulation at the time of delivery, France
Source: PLoS One. 2020 Oct 15;15(10):e0240782. doi: 10.1371/journal.pone.0240782 (PMC7561105; doi:10.1371/journal.pone.0240782)
Supplement: Attachment — Submitted filename: Dear Editor.docx [file pone.0240782.s004.docx]

Dear Editor,

PONE-D-20-20995

Based on reviewing the data from manuscript”Post lockdown COVID-19 seroprevalence and circulation at the time of delivery, France”, I made my comment below for your consideration.

The manuscript has analysed f 249 women for COVID-19 and found seroprevalence of SARS-CoV-2 was 8%. 47.4% of the SARS-CoV-2-IgG-positive pregnant women never experienced any symptoms. The author conclude that three weeks after the end of lockdown, SARS-CoV-2 infections were scarce . while a high proportion of SARS-CoV-2-IgG-negative pregnant women must be taken into consideration in the event of a resurgence of the pandemic in order to adapt public health measures to reduce exposure to the virus, such as social distancing and

teleworking for this specific population. The method is sound and the conclusion is trustable. I recommend publication of this paper in Plos One.

Best

Sincerely

Hengning Ke
